# Genetic Modifiers of Stroke in Patients with Sickle Cell Disease—A Scoping Review

**DOI:** 10.3390/ijms25126317

**Published:** 2024-06-07

**Authors:** Morohuntodun O. Oni, Miguel Brito, Chloe Rotman, Natasha M. Archer

**Affiliations:** 1 Pediatric Hematology/Oncology, Dana-Farber/Boston Children’s Cancer and Blood Disorders Center, Harvard Medical School, Boston, MA 02115, USA; morohuntodun.oni@childrens.harvard.edu; 2 H&TRC—Health & Technology Research Center, ESTeSL—Escola Superior de Tecnologia da Saúde, Instituto Politécnico de Lisboa, 1990-092 Lisbon, Portugal; miguel.brito@estesl.ipl.pt; 3 Medical Library, Boston Children’s Hospital, Boston, MA 02115, USA; chloe.rotman@childrens.harvard.edu

**Keywords:** sickle cell disease, genetic modifiers, stroke, cerebral vasculopathy

## Abstract

Sickle cell disease (SCD) clinically manifests itself with a myriad of complications. Stroke, both ischemic and hemorrhagic, as well as silent white matter changes, occurs at a relatively high prevalence. Understanding why and in whom stroke is most likely to occur is critical to the effective prevention and treatment of individuals with SCD. Genetic studies, including genome- and exome-wide association studies (GWAS and EWAS), have found several key modifiers associated with increased stroke/stroke risk in SCD via mechanisms including Hemoglobin F (HbF) modulation, inflammation, cellular adhesion, endothelial disruption, and hemolysis. We present a review on the modifiers that have most clearly demonstrated an association to date. More studies are needed to validate other potential polymorphisms and identify new ones. Incorporating gene-focused screenings in clinical care could provide avenues for more targeted, more effective, and less toxic prevention of stroke in this population. The data from this review will be used to inform the initial GWAS performed by the International Hemoglobinopathy Research Network (INHERENT) consortium.

## 1. Introduction

Sickle cell disease (SCD), a monogenic disease affecting blood rheology and endothelial function, clinically manifests itself with a myriad of vascular complications including stroke. The sickle hemoglobin (HbS) variant is caused by a valine–glutamine substitution and subsequent abnormal hemoglobin polymerization. Polymerization results in red blood cell (RBC) sickling and a dynamic cascade of events related to hemolysis, vaso-occlusion, and/or sterile inflammation. Hemolysis, through the depletion of nitric oxide, impairs endothelial dilatation and activates the endothelium, leading to the recruitment of leukocytes, platelet aggregation, and trapping of sickled erythrocytes, i.e., vaso-occlusion. Both hemolysis and vaso-occlusion contribute to sterile inflammation, a key feature of the disease. A plethora of downstream complications including severe anemia and vasculopathy can occur, resulting in end-organ damage.

Stroke, both ischemic and hemorrhagic, as well as silent white matter changes, occurs at a relatively high prevalence in patients with severe SCD [1]. The pathophysiology varies largely based on the kind of stroke (large vessel vs. small vessel; ischemic or hemorrhagic) but displays recurring themes. Sickled erythrocytes display increased adhesion characteristics to the vascular endothelium [2]. These erythrocytes—either due to hemolysis, increased adherence to the endothelium, or increased adherence to each other—encourage a pro-inflammatory environment that is susceptible to increased expression of cellular adhesion molecules, platelet aggregation, increased cytokines, pro-thrombotic activity, and eventually, vasculopathy and occlusion [3]. These can manifest as silent cerebral infarcts, overt ischemic stroke, and hemorrhagic stroke, with hemorrhagic stroke being more prevalent in adults and cerebral infarcts more common in pediatric populations. Understanding why and in whom stroke is most likely to occur is critical to the effective management and treatment of individuals with SCD. Genetic studies, including genome- and exome-wide association studies (GWAS and EWAS), have investigated several key modifiers associated with increased stroke and/or stroke risk (defined as conditional and/or abnormal transcranial Doppler velocities) in SCD based on the pathophysiology of the disease. Many of these genetic modifiers have been investigated based on their known phenotypic effects, e.g., decreased sickling as in those modifiers associated with fetal hemoglobin (HbF) induction or increased hemolysis, as is the case with glucose 6 phosphate dehydrogenase (G6PD) deficiency [4,5].

The International Hemoglobinopathy Research Network (INHERENT) studies the role of genetic modifiers in hemoglobinopathies and seeks to better understand how non-sickle-hemoglobin-related genes contribute to disease severity [6]. Using GWAS of clinically annotated, geographically diverse blood samples, the consortium aspires to both identify and validate single nucleotide polymorphisms (SNPs) that either enhance or diminish the pathophysiology associated with stroke, as well as many other complications associated with SCD.

Here, we performed a scoping review of genetic modifiers of stroke in SCD. We grouped the modifiers discussed into five main mechanistic categories: HbF modulation, inflammation, cellular adhesion, endothelial disruption, and hemolysis. Given the currently approved therapies for SCD management and treatment, including hydroxyurea, SNPs involving HbF-related genes have been the most frequently investigated and validated. While many polymorphisms still need to be confirmed, even more studies are needed to identify the many other potential polymorphisms that contribute to the variability in phenotypic expression associated with SCD. The data from this review will be used to inform the initial GWAS performed by the INHERENT consortium.

## 2. Methods

**Figure 1:**
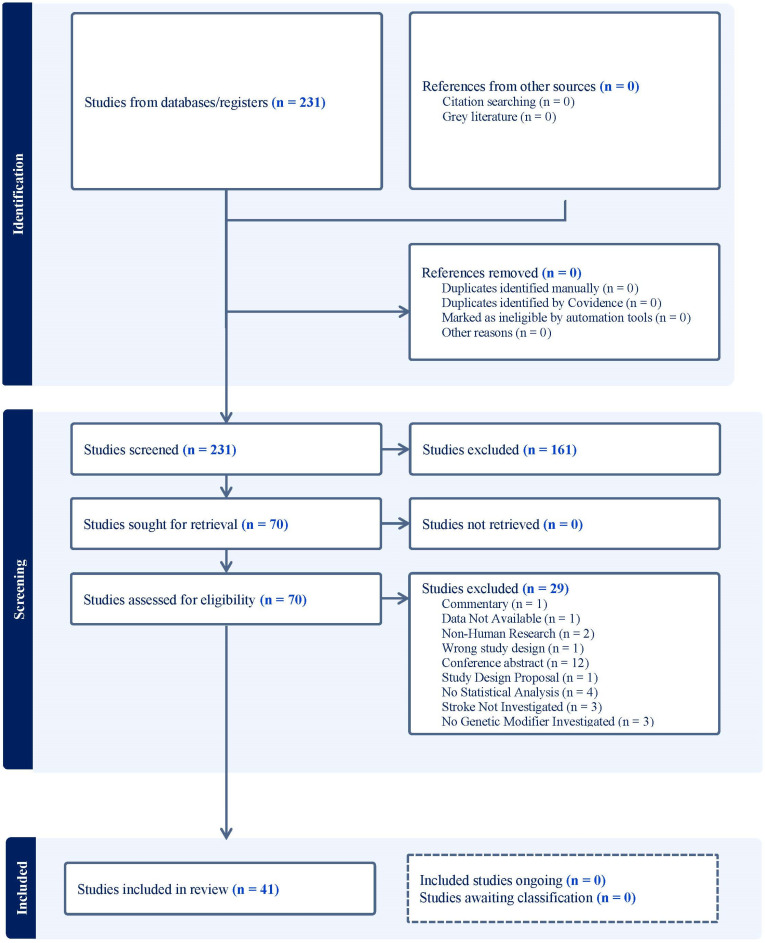
PRISMA table.

This review was designed following the PRISMA 2020 guidelines (Supplementary Table S1). The search was conducted in February 2023 by a medical librarian, using PubMed, EMBASE, and CINAHL. Developed between the medical librarian and clinicians, the search strategy included extensive keywords related to sickle cell anemia, stroke and similar cerebrovascular events, and relevant genetic markers. These include but are not limited to “genetic biomarkers”, “betaglycan”, “glucosephosphate dehydrogenase”, and “ANXA2 protein”. The full search strategies for all databases are available in Appendix A. Results were exported to EndNote and duplicated according to the method set forth by Bramer et al. and then uploaded to Covidence for screening [7]. Of the original 231 unique citations, 70 were assessed for full-text screening and 41 were included for data extraction (Figure 1). The protocol for this systematic review was registered on PROSPERO and can be accessed at https://www.crd.york.ac.uk/prospero/display_record.php?RecordID=397784.

### 2.1. Article Selection

Articles were reviewed and selected in 3 phases. Using the Covidence platform, 3 reviewers (M.O.O, N.M.A, and M.B) completed a review of the titles and abstracts of 231 articles. Titles and abstracts must have referenced stroke (including ischemic, hemorrhagic, or brainstem stroke, transient ischemic attack, or cerebral infarction), stroke risk (using transcranial Doppler velocities), or cerebral vasculopathy and SCD. Titles or abstracts that did not mention genes, genetic modifiers, and genetic conditions were excluded from this review. Once titles and abstracts were screened, 2 reviewers (M.O.O and M.B) read each full article and voted to include, exclude, or unsure. Articles were excluded if they were conference abstracts, commentaries, or systematic reviews, if they did not investigate a specific polymorphism or did not discuss stroke, if they did not have extractable data or did not conduct a statistical analysis, if the research was not conducted on humans, or if the study was not based on human research. Articles that met inclusion and exclusion criteria were discussed in a meeting with all 3 reviewers for consensus. Any final conflicts were resolved by a final reviewer (N.M.A).

### 2.2. Data Extraction

Data were extracted using a standardized list of variables including gene, SNP rs_ID, chromosome, base pair location, study type, cohort size, disease, trait investigated, effect allele frequency, and statistical analysis. Disease groups were sorted into patients with SCA—homozygous sickle cell (HbSS) and sickle beta 0 thalassemia (HbSB0thal)—and patients with SCD—any sickle disease genotype. Traits investigated were divided into 3 categories—stroke (defined as incidence of ischemic, transient ischemic attack, hemorrhagic, brain stem, silent cerebral infarction), stroke risk (defined as conditional or abnormal TCDs), and cerebral vasculopathy. Reviewers extracted data from 13 to 14 papers each. Finally, the extracted data were standardized for consistency.

## 3. Results

### 3.1. Search Results

Two hundred and thirty-one studies were identified using the search method described. One hundred and sixty-one papers were excluded in title and abstract review based on the inclusion/exclusion criteria detailed in the methods. Seventy papers were screened in manuscript review, and we were able to extract data from forty-one different papers and abstracts (Figure 1). The forty-one manuscripts reviewed consisted of cohort studies, case–control studies, studies using Whole Genome Sequencing (WGS) or GWAS analyses, and one functional study. The highest number of studies (41%) were conducted using US-based cohorts, including patients from the Cooperative Study of Sickle Cell Disease (CSSCD), Long Term Effects of Hydroxyurea Therapy in Children with Sickle Cell Disease (HUSTLE), and Stroke With Transfusions Changing to Hydroxyurea (SWiTCH) cohorts. Other cohorts included participants from Brazil, France, Nigeria, Sudan, Tanzania, Saudi Arabia, and Cameroon. Individuals with SCA (HbSS and HbS-Beta-Zero thalassemia) and more broadly SCD were included in the studies, with the majority (59%) covering all SCD genotypes. Population sizes ranged from 91 to 1333 participants, with the largest population coming from the Brazil REDS-III SCD cohort. Cohorts mainly consisted of participants identifying as having African ancestry.

In total, the 41 papers analyzed the association between 18 genes and 25 SNPs and stroke in patients with SCD (Table 1). Genes were separated into five mechanistic categories where 11% were HbF modulators, 33% were involved in inflammatory processes, 17% were involved in cellular adhesion, 28% were involved in endothelial disruption, and 11% were involved in hemolysis. Alpha thalassemia was the genetic modifier most reported on, present in 40% of the studies included. This was followed closely by BCL11A and G6PD.

### 3.2. Fetal Hemoglobin Induction

HbF values are monitored closely in clinical settings for their drastic effect on clinical and hematologic SCD severity and outcomes [26]. HbF, composed of two alpha- and two gamma-globin chains, disrupts the hemoglobin polymerization process which produces sickled erythrocytes [27]. The F-cells created have increased lifespans compared to sickled erythrocytes and are less prone to hemolysis [26]. These cells also have decreased adhesion properties, reducing the extent of the vaso-occlusion characteristic of sickled erythrocytes [3]. Overall, these lead to a decreased risk of severe anemia and ischemia, resulting in decreased stroke risk. As a result, HbF genetic modulators have been studied extensively for their role in decreasing vaso-occlusive episodes, decreasing hemolysis, and improving clinical outcomes. The most investigated modifiers of fetal hemoglobin include *HBG1/2, HBS1L-MYB,* and *BCL11A,* already the target in several gene therapy trials [28,29,30].

#### 3.2.1. BCL11A

The most referenced genetic modifier that causes HbF induction and is associated with decreased stroke and stroke risk in SCD is the *BCL11A* gene [31]. *BCL11A*, a transcriptional repressor of HbF, decreases HbS polymerization in vitro. Because of its significant suppressive effect on gamma-globin expression and HbF formation, the clinical manifestations of several *BCL11A* gene SNPs have been investigated.

The *BCL11A* rs1427407_T variant is the most widely investigated SNP as a potential genetic modifier of hemolysis and vaso-occlusion in SCD [8]. However, the T allele seems to be the minor allele in patients with African or African American ancestry, with allele frequencies observed between 0.25 and 0.29 [32]. The T allele has been associated with decreased clinical complications and increased HbF. However, Saraf et al. found no significant relationship between the rs1427407_T allele and stroke incidence; they did find a significant association between this variant and stroke in the absence of an α-thalassemia gene deletion [9]. Classifying SCA patients with either the rs1427407_T allele or α-thalassemia trait as “standard risk”, “high-risk” patients (with both no T allele and no α-thalassemia inheritance) were seen to have a significantly increased risk of stroke in a combined analysis of three separate cohorts (OR = 2.0; 95% CI = 1.33–2.99; *p* = 0.0002). Hassan et al. expanded on this and showed that the rs1427407_G variant in particular is associated with a higher frequency of recurrent stroke compared to the GT and TT phenotype (74.1% versus 50.6% and 57.6%, respectively, *p* < 0.05) [8].

Other *BCL11A* SNPs have been investigated, and while others are associated with increased HbF, such as rs1 1886868_C and rs4671393_A, they have yet to be investigated or validated as potential stroke modifiers [33].

#### 3.2.2. HBG2

SNPs of the gamma-globin gene *HBG2* were investigated in the same study as an HbF inducer and another potential genetic modifier of stroke. The rs7482144_A allele is significantly associated with increased HbF but has not yet been investigated as a stroke modifier to our knowledge [33].

#### 3.2.3. Other Fetal Hemoglobin Inducers

SNPs of other HbF inducers, including *HBS1L-MYB* and *HMIP*, have been investigated to lesser degrees, but a significant association with these genes and stroke has yet to be identified in SCD [34].

### 3.3. Inflammation

A study investigating patients who have experienced cerebral infarcts vs. controls observed increased levels of inflammatory cytokines and chemokines in patients with stroke [35]. The increased expression of these inflammatory markers is linked to the activation and expression of cellular adhesion molecules in leukocytes and endothelial cells, leading to occlusion [36]. White blood cells, when exposed to the pro-inflammatory environment, also experience increased cellular and vascular adhesion and aggregation, contributing to stroke risk. The inflammatory markers create a hyper-coagulable environment in the vessel, encourage endothelial cells to release vasoconstrictors, and downregulate vasodilator production [3]. The effects on vascular quiescence and vasomotor tone increase occlusion.

Increased platelet activity is also a marker of inflammation. Platelet activity markers have long been considered a significant indicator of stroke outcomes (particularly transient ischemic attacks). Platelet activity and reactivity can be higher in patients who have had ischemic strokes [37]. Though the mechanism of this relationship is not clear, studies hypothesize this relationship is due to platelet aggregation on thrombosis development. With patients with SCD, disease development results in not just sickled erythrocytes but the production of hyperactive platelets. These platelets overexpress pro-inflammatory and pro-thrombotic cytokines, have higher affinity to endothelial cells causing increased adhesion, and aggregate leading to occlusion and ischemia [38]. Antiplatelet medications—such as aspirin—have been implemented in several clinical sites for patients displaying frequent vaso-occlusion and patients who have had a stroke [39]. Polymorphisms related to platelet activity and reactivity in SCD could be significant modifiers of stroke in this population.

#### 3.3.1. TNF-α

While Tumor Necrosis Factor Alpha (TNF-α) was originally investigated for is anti-tumor properties, its role in inflammatory processes as well as the regulation of adhesion molecule expression on the vascular endothelium has now been widely documented [40]. It is proposed that the rs1800629_G variant, located in the promoter region of the *TNF-α* gene, is associated with the overexpression of this pro-inflammatory cytokine; however, this association has yet to be validated [41]. Belisaro et al. demonstrated that this variant increases the risk of stroke in patients with SCA (OR 2.71, 95% CI 1.29–6.04; *p* = 0.009) [14]. Hoppe et al. in 2003 reviewed the same variant in patients with SCA in the CSSCD cohort and found the variant to indicate increased stroke risk (OR 0.52, SE 0.17; *p* = 0.048) in large-vessel strokes and confirmed their findings 3 years later in a reported risk analysis on 96 patients with SCA (OR = 3.27; 95% CI = 1.6, 6.9; *p* = 0.006) [12,13]. The same study found the rs1800629_A allele to be protective of large-vessel stroke in the same cohort (OR = 0.39; 95% CI = 0.19, 0.76; *p* = 0.006).

#### 3.3.2. TGFβR-3

The transforming growth factor beta receptor 3 gene is one in a series of TGF-β receptors and is noted as one of the key receptors in various inflammatory signal transduction pathways [42]. A recent study suggests that the concentration of TGF-β receptors can be indicative of steady state or crisis in pediatric populations [42]. Belisaro et al. found that within their SCA cohort of 395 pediatric patients, having at least one rs284875_A allele was associated with high-risk TCD but not associated with acute cerebral ischemia (HR = 3.37; 95% CI = 1.59, 7.12; *p* = 0.0015) [15]. This observed relationship supports findings in a predictive Bayesian model, which did not fall under the scope of this review [43], but also showed that the rs284875_A variant was predictive of higher TCDs. Research conducted by Flanagan et al. in their case–control analysis investigating 233 patients from the HUSTLE and SWiTCH cohorts also investigated rs284875. Their analysis found that the homozygous and heterozygous presence of the rs284875_T allele was indicative of higher stroke risk compared to the C allele (OR = 2.53; 95% CI = 1.26, 4.99; *p* = 0.005) [10]. There has been no direct comparison made between the A and T alleles of this SNP.

#### 3.3.3. LTC4S

Patients with SCD have elevated cysteinyl leukotrienes, a pro-inflammatory marker and mediator of vasoconstriction, and upregulation of cellular adhesion in the vasculature [44]. Leukotriene C4 synthase (LTC_4_S) is a key enzyme involved in the genesis of leukotrienes, and the rs730012_C variant has been associated with the overexpression of this synthase. Hoppe et al.’s pediatric SCA cohort found *LTC_4_S* to be protective of large-vessel stroke risk in their African American population (OR = 0.39; 95% CI = 0.17, 0.19; *p* = 0.03) [13].

#### 3.3.4. IL4R

In its role as an inflammatory cytokine, Interleukin-4 (IL4) plays a major role in endothelial adhesion to lymphocytes and is believed to work with TNF and IFN-y (interferon-gamma) in exacerbating chronic inflammation [45]. The rs1805015_C missense variant of the IL4 receptor results in a serine/proline substitution. The resulting receptor was hypothesized to be associated with increased risk of stroke, deduced from Hoppe et al.’s 2004 investigation of the CSSCD cohort (OR = 2.5; 95% SE = 0.83; *p* = 0.006) [12]. However, their confirmation analysis using pediatric patients found this association to only be approaching significance (OR = 1.6; 95% CI = 0.93, 2.8; *p* = 0.09) [13].

#### 3.3.5. ADCY9

Adenylyl cyclase 9 is a key enzyme involved in the generation of cAMP from ATP. Highly expressed in the brain, ACDY9 plays a key role in signaling cascades in the CNS [46]. In their SCA cohort, Flanagan et al. found the *ACDY9* rs2238432_A variant to be associated with decreased incidence of stroke (OR = 0.47; 95% CI = 0.28–0.79; *p* = 0.003) [10].

#### 3.3.6. GOLGB1

In the context of platelet activity, the Golgin B1 (*GOLGB1*) gene, which plays a role in platelet activation and maintenance of the actin cytoskeleton, is also responsible for creating the coat protein 1 in the Golgi apparatus. Analysis of the rs3732410_C (tyrosine to cysteine) variant in macrophages found the variant to be associated with a more structurally sound Golgi apparatus and lower levels of proteins associated with platelet activation as well [47]. This could provide further insight into why, in a cohort of 677 patients with SCA, Flanagan et al. found the allele to be protective against stroke (OR = 0.17; 95% CI = 0.06–0.42; *p* < 0.001) [11].

### 3.4. Cellular Adhesion

Cellular adhesion molecules (CAMs), including selectins, cadherins, and integrins, have been investigated extensively for their relationship with stroke. Several studies investigating persons who have and have not had a stroke have found significant relationships between serum CAM levels and ischemic stroke [48,49]. Increased CAM expression on cells and in the vascular endothelium encourages increased leukocyte adhesion and cellular aggregation, encouraging ischemia in the cerebral vasculature and leading to stroke risk. Increased inflammation in patients with SCD promotes CAM expression in leukocytes and endothelial cells. The resulting increased CAM expression increases occlusion and ischemia risk in patients. This has been believed to contribute to the increasing stroke risk of patients with more severe SCD-related inflammation.

#### 3.4.1. TEK

The TEK receptor tyrosine kinase regulates angiogenesis and maintains vascular quiescence. It also prevents the leakage of pro-inflammatory plasma proteins and leukocytes from blood vessels [50]. The *TEK* rs489347 SNP encodes a tyrosine kinase expressed on endothelial cells. Flanagan et al. found the rs489347_G allele to be associated with increased stroke risk using a recessive genetic model (where for an allele A, genotypes are grouped as AA vs. Aa and aa) (OR 2.16, 95% CI 1.11–4.23) [10]. A similar analysis conducted on patients with SCA in a Brazilian cohort yielded comparable findings. Belisario et al. found the rs489347_C variant to be associated with increased risk of cerebral ischemia (OR 3.05, 95% CI 1.28–7.29; *p* = 0.012) [15].

#### 3.4.2. VCAM-1

Vascular cell adhesion molecule 1 (VCAM-1) is a key cellular adhesion molecule that is expressed on the vascular endothelium. VCAM-1 plays a significant role in facilitating the inflammatory response, adhering sickle erythrocytes, leukocytes, and other cells to the endothelium. As a result, it is highly suspected to be a key genetic indicator of stroke in patients with SCD. Taylor et al. investigated several *VCAM-1* variants and their relationship with clinical symptoms of SCD. Their cohort of 102 patients with HbSS proposed the rs3783613_C allele to be protective of stroke (OR = 0.35; 95% CI 0.15–0.83; *p* = 0.02) [17]. Another study, however, did not find a significant relationship between this SNP and stroke risk [14]. Hoppe et al. saw the opposite association with the rs1041163_C allele and found the variant to be associated with the increased incidence of small vessel stroke in their cohort of 230 SCD patients (OR = 1.98; 95% SE = 0.43; *p* = 0.0002) [12].

Silva et al. also explored the influence of genetic modifiers on cerebral vasculopathy and found that the *VCAM-1* rs1409419_T allele was associated with elevated TCD velocity and stroke events in pediatric patients with SCA (OR = 4.17; 95% CI 1.57–14.13; *p* = 0.009) (OR = 4.71; 95% CI 1.04–16.73; *p* = 0.041) [16]. This same study found the same association with haplotype 7 of the *VCAM-1* promoter region, which has been associated with increased hemolysis in patients with SCA (OR = 4.17; 95% CI 1.57–14.13; *p* = 0.009).

#### 3.4.3. ITGA4

Integrin subunit alpha 4 is a binding receptor that plays a role in sickle erythrocytes and white blood cell adhesion to the vascular endothelium. The rs113276800_A and rs3770138_T variants of this receptor were found to be positively associated with stroke in pediatric patients with SCA (OR = 7.62; 95% CI 1.39–41.65; *p* = 0.025) (OR = 5.57; 95% CI 1.12–27.67; *p* = 0.045) [16].

### 3.5. Endothelial Disruption

Sickled erythrocytes tend to have significantly increased adherence to the vascular endothelium [3]. Dissociating the two from one another requires significantly increased shear stress that, once disconnected, results in significant mechanical injury to the endothelial cells [51]. Repairing these endothelial tears increases vasomotor tone and occlusion. Moreover, this disruption is associated with an influx of inflammatory and pro-thrombotic agents to maintain vascular quiescence but inevitably also increase vaso-occlusion. Notably, endothelial cells are key producers of NO [52]. The decrease in NO synthesis from this injury also contributes to decreased vasodilation and increased vaso-occlusion. As a result, genetic modifiers of endothelial cell activity could be significant modifiers of stroke.

#### 3.5.1. ANXA2

Annexin-A2 (ANXA2) is a plasminogen receptor on the vascular endothelium and is also associated with surface plasmin generation and hyper-coagulation in SCA [53]. *ANXA2* variants have been associated with unregulated plasmin activity leading to cerebrovascular events. In 233 pediatric patients with SCA, the rs11853426_T was positively associated with the incidence of stroke (OR = 2.7; 95% CI 1.25–5.84; *p* = 0.007) [10].

#### 3.5.2. ENPP1

The ectonucleotide pyrophosphatase/phosphodiesterase 1 (*ENPP1)* gene provides instructions for making the protein ENPP, which helps break down extracellular adenosine triphosphate (ATP) into adenosine monophosphate (AMP) and pyrophosphate. Pyrophosphate helps prevent the accumulation of calcification and mineralization in the body. In 2013, Flanagan et al.’s analysis of the *ENPP1* rs1044498_C variant—resulting in a glutamine instead of lysine residue—predicted decreased stroke risk (OR = 0.49; 95% 0.35–0.69; *p* < 0.001) [11]. The glutamine residue has been demonstrated to increase enzyme levels and activity. Belisaro’s Brazilian cohort in 2015, however, found that the variant rs1044498_A indicated an increased stroke risk (*p* = 0.011) for 395 patients [14]. Silva et al. noted the same exacerbating effect in their analysis of the rs1044498_A allele (OR = 4.03; 95% CI = 1.21–13.42; *p* = 0.026) [16]. However, this cohort reported similar minor allele frequencies between their cohort and other African ancestry cohorts.

#### 3.5.3. NOS3

The nitric oxide synthase 3 (*NOS3*) gene has also been investigated as a stroke modifier due to its role in maintaining NO levels during episodes of hemolysis and oxidative stress. Silva et al. investigated several variants of the *NOS3* gene and its relationship with stroke risk, cerebral vasculopathy, and silent cerebral infarctions in 70 SCA patients with sub-Saharan African ancestry [16]. In this cohort, they found the VNTR and haplotype V variants to be protective of stroke risk (OR = 0.1; 95% CI = 0.01–0.69; *p* = 0.03; OR = 0.18; 95% CI = 0.04–0.81; *p* = 0.031) and the haplotype VII to be protective of cerebral vasculopathy (OR = 0.08; 95% CI = 0.01–0.69; *p* = 0.006).

#### 3.5.4. CBS

Of all the potential modifiers of stroke investigated, the ones associated with homocysteine metabolism are amongst the least investigated. Cystathionine Beta Synthase (CBS) is a key enzyme in the homocysteine metabolic pathway. Hyperhomocysteinemia (HHcy) has been associated with an increase in inflammatory cytokines, thrombotic damage, and resulting degradation of the vascular endothelium. It has been observed consistently in patients who have had ischemic strokes [54]. In a cohort of 69 patients, Hoppe et al. found the rs5742905_G variant of the *CBS* gene to be associated with a decreased risk of stroke (OR 0.32; 95% CI 0.9–5.5; *p* = 0.03) in patients with SCA [18].

#### 3.5.5. MTHFR

Methylenetetrahydrofolate reductase (MTHFR) has also been investigated as a risk factor for vascular complications in patients with SCD. In their analysis, Neto et al., 2006, investigated the *MTHFR* C677T variant in 53 Brazilian patients through the lens of its role in vascular injury, where they observed no significant association between the rs1016843877_T variant and stroke, retinopathy, acute thoracic syndrome, or avascular necrosis [19]. They did, however, identify a significant association between the variant and increased risk of cerebral vasculopathy (described as stroke, retinopathy, and avascular necrosis) (OR 3.5; 95% CI 1.1–11.5; *p* ≤ 0.05), which they attributed to HHcy in patients with this variant, though homocysteine levels were not collected.

### 3.6. Hemolysis

One of the most notable consequences of SCD is the increased hemolytic rate. Hemolysis produces free hemoglobin, producing a cascade of downstream effects [55]. Firstly, the free heme causes oxidative stress in the vessel, and in its form, acts as a nitric oxide (NO) scavenger. The NO concentration is paramount to the maintenance of the tone of the vascular endothelium since NO plays a key role in vasodilation. NO depletion increases vascular tone, leading to vaso-occlusion and ischemia. The free heme also triggers the upregulation of pro-inflammatory genes, releasing cytokines and causing reactive oxidative species expression, the effects of which create increased vaso-occlusion. As a result, genes that regulate the hemolytic rate and/or free heme production in SCD could be instrumental modifiers of stroke.

#### 3.6.1. Alpha Thalassemia Trait

The alpha thalassemia trait is a commonly inherited mutation in *HBA1/HBA2* genes which is associated with increased hemoglobin values in adults with SCD. The most prevalent deletions are the −α^3.7^ and −α^4.2^ deletions in the *HBA* genes. The −α^3.7^ deletion is commonly observed in cohorts with African, sub-Saharan, and Asian ancestry, with 30% of black patients lacking one alpha-globin gene (alpha thal-2 genotype: aa/a-) and 4% lacking two genes (alpha thal-1 genotype: a-/a- or, rarely, aa/--) [56]. Trait carriers usually present as hypochromic and chronically microcytic but tend to be significantly protected from developing malaria [57].

In 1994, Adams et al. found a significant difference in α-thalassemia inheritance in patients with stroke compared to those without (38% vs. 20.5%) [58]. For the 300 SCA patients, this study indicated that α-thalassemia is protective of stroke; however, the two cohorts also had significantly different HbF values. The team called for further investigation of this relationship in larger cohort studies.

Several studies have stemmed from these findings. Hsu et al. found children with SCD and α-thalassemia to be four times more likely to have normal TCD results (OR = 4.1; 95% CI = 2.2–7.7; *p* < 0.0001) [59]. Bernaudin et al. saw even higher protective effects for patients with SCA (OR = 6.45; 95% CI = 2.21–18.87) [20]. Belisário et al. looked at cerebrovascular disease (overt ischemic stroke, abnormal TCD, transient ischemic attack, and abnormal cerebral angiography) and concluded that the α-thalassemia trait protected individuals with SCA from cerebrovascular disease, with the effect being equally protective for both HbSS and HbSβ0 thalassemia. Cox et al. found this consistent in both cis and trans α-thalassemia carriers in a large scale—601—SCA patient cohort. Various subsequent studies validated these findings [20,21,60].

Consistent findings resulted in researchers investigating whether co-inheritance of α-thalassemia and other genetic modifiers would be more predictive of stroke, stroke risk, and cerebral vasculopathy, particularly investigations on co-inheritance with certain *BCL11A* variants (discussed earlier) and MCS-R2 variants. The *MCS-R2* rs11865131_A variant seems to eliminate the α-thalassemia protective effect, while the effect is retained in the GG genotype (OR = 1.29; 95% CI = 0.65–2.58) vs. (OR = 0.29; 95% CI = 0.14–0.6); *p* = 0.0049 [22].

#### 3.6.2. G6PD

Glucose-6-phosphate dehydrogenase (G6PD) deficiency is an inherited genetic disorder in which G6PD, which generates NADPH and protects RBCs from oxidative injury, is decreased. Individuals with G6PD deficiency can have increased hemolysis when exposed to oxidative stress. In addition, vascular injury may be worsened in individuals with both G6PD deficiency and SCD due to decreased nitric oxide, which typically stimulates endothelial cell proliferation. In 2008, Bernaudin et al. found that G6PD deficiency (as demonstrated by NADPH activity) was associated with an increased risk of abnormal TCD (OR = 2.54; 95% CI 1.12–5.35; *p* = 0.024) [20]. This association was observed again in a cohort of 516 participants with SCA, where the presence of any G6PD variant was associated with vasculopathy as defined by abnormal MRAs (OR = 2.78; 95% CI 1.04–7.42; *p* = 0.04) [23]. This was reinforced further in a study with 232 French pediatric patients with SCA (OR = 0.25; *p* < 0.01) [24]. Finally, in a Saudi Arabian population, the rs503086_T variant was associated with an increased risk of stroke in 48 patients with SCD compared to controls (OR = 12.5; 95% 1.76–113.7; *p* = 0.002) [25].

## 4. Discussion

The findings from this review outline the work already conducted to investigate the genetic modifiers of stroke in SCD but also help to identify potential future directions. As of this review, primary prevention through annual TCD screening has been the key preventer of stroke in the pediatric SCD population. The National Heart, Lung, and Blood Institute recommends patients aged 2–16 (or until TCD windows have closed) have annual TCD velocity screens to assess stroke risk [61]. For patients with persistently elevated velocities, primary prophylaxis through chronic transfusions has been the main line of defense against stroke incidence [62]. However, knowledge obtained from this review could provide an opportunity to investigate more targeted and efficient stroke screening by categorizing patients with certain genetic modifiers into risk-based cohorts that allow for more focused monitoring and risk management.

As of the time of this review, the investigations conducted on α-thalassemia have identified and validated trait inheritance as a significant indication of decreased stroke risk. In contrast, G6PD deficiency co-inheritance has been validated as an indicator of increased stroke risk. Furthermore, investigating the effects of inheriting several validated SNPs observed in high frequencies in patients with African ancestry could be used to further stratify these cohorts. For example, the rs1427407_T *BCL11A* variant when inherited with −α3.7kb significantly decreased the stroke risk in several cohorts [9]. Screening for these genes might be particularly helpful in determining whom to monitor for stroke with other imaging, such as MRI/MRAs, particularly once TCD screening windows have closed.

Future research might also focus on modifiers that decrease or increase the rate of hemolysis similarly to the α-thalassemia trait and G6PD deficiency, respectively. For α-thalassemia, the protective effect seems to be due to the biology of erythrocytes formed. Due to the α-globin deletion(s), individuals with this inheritance synthesize less overall hemoglobin and consequently decreased MC-HbS in each erythrocyte. The hemoglobin molecules undergo less polymerization, resulting in decreased hemolysis, improved red cell viability, and reduced severity of disease [63]. SNPs involved in or around alpha-globin formation, such as *MCS-R1*–*MCS-R4*, should be investigated more diligently, as the formation of hemoglobin seems to play a significant role in downstream stroke risk [22]. Genetic modifiers of hemolysis product scavengers like heme oxygenase 1 could also be potential targets for future research.

Genes involved in inflammatory responses regulate aspects of the pathophysiology of stroke in SCD [38]. Cytokines like TNFa and LTC4S and receptors like IL4R and TGFBR3 act as the spark for the inflammatory responses leading to cerebral vasculopathy like increased hemolysis, endothelial disruption, and vaso-occlusion. As a result, a comprehensive review of genes involved in the pro-inflammatory response and their relationship with stroke could be an important contribution to the literature and to the establishment of stroke risk cohorts. These should be considered high-priority, as a pro-inflammatory environment regulates many of the other genetic variables shown to exacerbate stroke risk.

Modifiers of CAM activation and expression also demonstrate another potential genetic modifier that can be screened for or incorporated into clinical evaluation. Intercellular Adhesion Molecule 1 (ICAM-1), VCAM-1, P-selectin, and E-selectin are the four most cited CAMs expressed at high levels for patients with increased stroke risk. While this analysis yielded *VCAM-1* polymorphisms as significant modifiers, more research needs to be conducted on P- and E-selectins or *ICAM-1*. Further research on these could provide key insight into their role as modifiers of stroke incidence and stroke outcomes in SCD given their effect on stroke outcomes in other cohorts [64]. Several polymorphisms in this review were also related to NO bioavailability and efficacy. Given the significant effect NO concentration seems to have on SCD pathophysiology and stroke development, it should be treated as a more important modifier of disease severity. More should be invested into the identification and validation of *NOS3* and other endothelial *NOS* polymorphisms, especially the *VNTR*, *haplotype V*, and *haplotype VII* polymorphisms seen in patients with SCD [65,66,67].

In the wake of the approved advanced therapy medicine products that encompass gene therapy, it is important to note the role such therapies might have in alleviating stroke risk. Both FDA-approved therapies for SCD (Casgevy—modification using CRISPR-Cas9 to knockdown *BCL11A*; Lyfgenia—the addition of a modified form of beta-globin to produce HbA^T87Q^, a non-sickling hemoglobin) were based on trials that excluded patients with any history of cerebral vasculopathy and even those on primary prophylaxis for stroke risk. Several studies reviewed in this cohort validated the association between the rs1427407_T variant that suppresses *BCL11A* and stroke risk. This very same variant’s modulating effect on HbF levels was the basis for many of the gene therapies now being administered to this population. Approved gene therapy suppressing *BCL11A* could be significant in preventing stroke development, particularly in those with identified increased stroke risk. Other HbF related genes, including *KLF1*, *HBS1L-MYB*, *SOX6*, *p22NF-E4*, *COUP-TFII*, *DRED*, and *FOP*, which could be significant modulators of SCD outcomes including stroke risk, should be further investigated as potential gene therapy targets [68].

### 4.1. INHERENT

There is a need for large-scale, genome-wide investigation of patients with SCD to better classify genetic modifiers of stroke. The largest cohort in our investigation pooled data from 1333 SCD participants in three different centers. The sample repository and data registry generated by the INHERENT will provide not only significantly more patients from whom data can be analyzed but will also provide ethnic diversity in the cohort resulting in opportunities for national and global genetic data.

### 4.2. Limitations

Despite trying to review all current research covering genetic modifiers of stroke in the SCD population, our search may have missed articles contributing key information if they did not fall within our strict search criteria (such as English text) and limited number of databases searched. This is highlighted by the fact that a large proportion of our studies were conducted in the US. Another important criterion for our review was the presence of a study statistic, which may have limited our data pool. We also chose stroke, stroke risk, and cerebral vasculopathy as relevant outcomes, but these key terms are very specific and it may have been helpful to widen our search to include all brain-related outcomes. Lastly, the search completed for this analysis was conducted in February 2023 and does not contain data published since then.

## 5. Conclusions

While the literature has shown significant relationships between certain SNPs and stroke risk in patients with SCD, more research needs to be conducted to validate further suspected genes and implement these findings into patient care. Stroke is one of the most devastating risks for patients with SCD, and the early assessment of risk is paramount for successful intervention in this patient population. Incorporating gene-focused targeted screening of patients with SCD could improve the disease monitoring and management of high-risk patients. Incorporating these findings in clinical care could provide avenues for more targeted and less toxic prevention of stroke in this population. Moreover, the current curative therapies approved in SCD could significantly benefit those with a genetic risk of stroke.

## Figures and Tables

**Table 1:** SNPs significantly associated with stroke risk.

Gene	SNP rsID/Common Name	Effect Allele	Allele Frequency	Effect Size	Observed Effect on Stroke Risk	Citation
Fetal Hemoglobin Induction						
*BCL11A*	rs1427407	G	0.74 *	IR = 74%	Increase	[8]
*BCL11A + Del −α3.7kb*	rs1427407	T	0.25 *	OR = 2.0	Decrease	[9]
Inflammation						
*ACDY9*	rs2238432	A	0.19 *	OR = 0.47	Decrease	[10]
*GOLGB1*	rs3732410	C	0.07 *	OR = 0.17	Decrease	[11]
*IL4R*	rs1805015	C	0.37 *	OR = 2.5 OR = 1.6	Increase	[12] [13]
*LTC4S*	rs730012	C	0.05 *	OR = 0.39	Decrease	[13]
*TNFa*	rs1800629	G	0.88 *	OR = 2.7 OR = 0.52 OR = 3.3	Increase	[14] [12] [13]
*TNFa*	rs1800629	A	0.12 *	OR = 0.39	Decrease	[13]
*TGFβR-3*	rs284875	A	0.01	HR = 3.4	Increase	[15]
*TGFβR-3*	rs284875	T	0.043	OR = 2.5	Increase	[10]
Cellular Adhesion						
*ITGA4*	rs113276800	A	0.06 *	OR = 7.6	Increase	[16]
*ITGA4*	rs3770138	T	0.06 *	OR = 5.6	Increase	[16]
*TEK*	rs489347	G	0.42	OR = 2.2	Increase	[10]
*TEK*	rs489347	C	0.75 *	OR = 3.1	Increase	[15]
*VCAM1*	rs1041163	C	0.19 *	OR = 1.98	Increase	[12]
*VCAM1*	Haplotype 7			OR = 4.2	Increase	[16]
*VCAM1*	rs1409419	T	0.40 *	OR = 4.2 OR = 4.7	Increase	[16]
*VCAM1*	rs3783613	C	0.03 *	OR = 0.35	Decrease	[17]
Endothelial Disruption						
*ANXA2*	rs11853426	T	0.37 *	OR = 2.7	Increase	[10]
*CBS*	rs5742905	G	0.02 *	OR = 0.32	Decrease	[18]
*ENPP1*	rs1044498	C	0.74 *	OR = 0.49	Decrease	[11]
*ENPP1*	rs1044498	A	0.26 *	OR = 4.0	Increase	[16]
*MTHFR*	rs1016843877	T	0.34	OR = 3.5	Increase	[19]
*NOS3*	VNTR **			OR = 0.10	Decrease	[16]
*NOS3*	rs2070744	C	0.15 *	OR = 0.18	Decrease	[16]
*NOS3*	rs1799983	T	0.13 *	OR = 0.080	Decrease	[16]
Hemolysis						
*Del −α3.7kb*	N/A	N/A	0.244 *	OR = 4.1 OR = 6.5	Decrease	[20] [21]
*Del −α3.7kb + MCS-R2*	rs11865131	A	0.18 *	OR = 1.3	Increase	[22]
*G6PD*	rs503086	T	N/A	OR = 2.5 OR = 2.8 OR = 0.25 OR = 12.6	Increase	[20] [23] [24] [25]

* Allele frequencies as reported in the NIH dbSNP accessed on 15 November 2023. ** Intron variable number of tandem repeats (VNTRs) 4B/4A. OR, odds ratio; IR, incidence ratio; HR, hazard ratio; N/A, not applicable.

## Supplementary Materials

The following supporting information can be downloaded at: https://www.mdpi.com/article/10.3390/ijms25126317/s1

## Appendix A

PubMed	(“anemia, sickle cell”[mesh] OR “sickle cell anemia” OR “sickle cell anaemia” OR “sickle anemia” OR “sickle anaemia” OR drepanocytosis OR “hemoglobin s disease” OR “sickle cell disorder” OR “sickling disorder” OR “hbs disease” OR “sickle cell disease”) AND (stroke[mesh] OR stroke OR strokes OR “Cerebrovascular Accident” OR “Cerebrovascular Accidents” OR Cerebrovascular Apoplexy OR CVA OR CVAs OR “Brain Vascular Accident” OR “Brain Vascular Accidents” OR “brain attacks” OR “brain accidents” OR “brain insult” OR “brain insults” OR “cerebral attack” OR “cerebral attacks” OR “cerebral accident” OR “cerebral accidents” OR “cerebral insult” OR “cerebral insults” OR “cerebral vascular insufficiency” OR “cerebrovascular attack” OR “cerebrovascular attacks” OR “cerebrovascular accident” OR “cerebrovascular accidents” OR “cerebrovascular insult” OR “cerebrovascular insults”) AND ((“genetic markers”[mesh] OR “genetic marker” OR “genetic markers” OR “genetic biomarker” OR “genetic biomarkers” OR “dna marker” OR “dna markers” OR “chromosome marker” OR “chromosome markers”) OR (BCL11A) OR (betaglycan[supplementary concept] OR TGFBR3 OR “beta-glycan” OR betaglycan OR “TGF-beta type III” OR “TGF-beta receptor type III” OR “transforming growth factor beta type III” OR VCAM1) OR (“adenylate cyclase 9”[supplementary concept] OR “adenylate cyclase 9” OR “adenylyl cyclase IX” OR ADCY9 OR “adenylyl cyclase type IX”) OR (“ANXA2 protein, human”[supplementary concept] OR ANXA2 OR “annexin A2 protein” OR “annexin 2”) OR (“TEK protein, human”[supplementary concept] OR TEK OR “TIE-2” OR tie OR “angiopoietin-1 receptor” OR “angiopoietin receptor”) OR (“adenylate cyclase 9”[Supplementary Concept] OR “adenylate cyclase 9” OR “adenylyl cyclase IX” OR “adenylyl cyclase type IX” OR ADCY9) OR (“Vascular Cell Adhesion Molecule-1”[mesh] OR “Vascular Cell Adhesion Molecule 1” OR “Inducible Cell Adhesion Molecule 110” OR “CD106 Antigens” OR “VCAM 1” OR “CD106 antigen” OR “Vascular Cell Adhesion Molecule” OR “INCAM 110”) OR (“ectonucleotide pyrophosphatase phosphodiesterase 1”[Supplementary Concept] OR “ectonucleotide pyrophosphatase phosphodiesterase 1” OR ENPP1 OR “ecto-nucleotide pyrophosphatase phosphodiesterase 1” OR “glycoprotein PC-1” OR “plasma cell membrane glycoprotein PC-1” OR “alkaline phosphodiesterase 1” OR NPP1 OR “major aFGF-stimulated phosphoprotein OR “PC-1 glycoprotein” OR “plasma-cell membrane glycoprotein 1”) OR (“Glucosephosphate Dehydrogenase”[mesh] OR “plasma-cell membrane glycoprotein 1” OR “Glucosephosphate Dehydrogenase” OR “6 phosphate dehydrogenase” OR “6 phosphoglucose dehydrogenase” OR “Glucose 6 Phosphate Dehydrogenase”) OR (“alpha-Thalassemia”[mesh] OR “alpha-Thalassemia” OR “alpha thalassaemia) OR “Thalassemia alpha” OR “a thalassemia” OR “Hemoglobin H Disease”) OR (hbg2) OR (“BCL11A protein, human”[Supplementary Concept] OR BCL11A OR “C2H2-type zinc finger protein” OR “B-cell CLL-lymphoma 11A”))
EMBASE	(“sickle cell anemia”/exp OR “sickle cell anemia” OR “sickle cell anaemia” OR “sickle anemia” OR “sickle anaemia” OR drepanocytosis OR “hemoglobin s disease” OR “sickle cell disorder” OR “sickling disorder” OR “hbs disease” OR “sickle cell disease”) AND (“cerebrovascular accident”/exp OR stroke OR strokes OR “Cerebrovascular Accident” OR “Cerebrovascular Accidents” OR Cerebrovascular Apoplexy OR CVA OR CVAs OR “Brain Vascular Accident” OR “Brain Vascular Accidents” OR “brain attacks” OR “brain accident” OR “brain blood flow disturbance” OR “brain blood flow disturbances” OR “brain accidents” OR “brain insult” OR “brain insults” OR “cerebral attack” OR “cerebral attacks” OR “cerebral accident” OR “cerebral accidents” OR “cerebral blood flow disturbance” OR “cerebral blood flow disturbances” OR “cerebral insult” OR “cerebral insults” OR “cerebral vascular insufficiency” OR “cerebrovascular attack” OR “cerebrovascular attacks” OR “cerebrovascular accident” OR “cerebrovascular accidents” OR “cerebrovascular blood flow disturbance” OR “cerebrovascular blood flow disturbances” OR “cerebrovascular insult” OR “cerebrovascular insults”) AND ((“genetic marker”/exp OR “genetic marker” OR “genetic markers” OR “genetic biomarker” OR “genetic biomarkers” OR “dna marker” OR “dna markers” OR “chromosome marker” OR “chromosome markers”) OR (BCL11A) OR (“transforming growth factor beta receptor 3”/exp OR TGFBR3 OR “beta-glycan” OR betaglycan OR “TGF-beta type III” OR “TGF-beta type 3” OR “TGF-beta receptor type III” OR “TGF-beta receptor type 3” OR “transforming growth factor beta type III” OR “transforming growth factor beta type 3” OR VCAM1) OR (“adenylate cyclase 9” OR “adenylyl cyclase IX” OR ADCY9 OR “adenylyl cyclase type IX”) OR (ANXA2 OR “annexin A2 protein” OR “annexin 2”) OR (“angiopoietin receptor”/exp OR TEK OR “TIE-2” OR tie2 OR “angiopoietin-1 receptor” OR “angiopoietin receptor”) OR (“adenylate cyclase 9” OR “adenylyl cyclase IX” OR “adenylyl cyclase type IX” OR ADCY9) OR (“Vascular Cell Adhesion Molecule 1”/exp OR “Vascular Cell Adhesion Molecule 1” OR “Inducible Cell Adhesion Molecule 110” OR “CD106 Antigens” OR “VCAM 1” OR “CD106 antigen” OR “Vascular Cell Adhesion Molecule” OR “INCAM 110”) OR (“ectonucleotide pyrophosphatase phosphodiesterase 1” OR ENPP1 OR “ecto-nucleotide pyrophosphatase phosphodiesterase 1” OR “glycoprotein PC-1” OR “plasma cell membrane glycoprotein PC-1” OR “alkaline phosphodiesterase 1” OR NPP1 OR “major aFGF-stimulated phosphoprotein” OR “PC-1 glycoprotein” OR “plasma-cell membrane glycoprotein 1” OR “MAPF protein” OR “major acidic fibroblast growth factor-stimulated phosphoprotein”) OR (“glucose 6 phosphate dehydrogenase”/exp OR “plasma-cell membrane glycoprotein 1” OR “Glucosephosphate Dehydrogenase” OR “6 phosphate dehydrogenase” OR “6 phosphoglucose dehydrogenase” OR “Glucose 6 Phosphate Dehydrogenase” OR “6 phosphoglucoside dehydrogenase”) OR (“alpha Thalassemia”/exp OR “alpha-Thalassemia” OR “alpha thalassaemia” OR “Thalassemia alpha” OR “a thalassemia” OR “Hemoglobin H Disease”) OR (hbg2) OR (BCL11A OR “C2H2-type zinc finger protein” OR “B-cell CLL-lymphoma 11A” OR “CTIP1 protein”))
CINAHL	(DE “anemia, sickle cell” OR “sickle cell anemia” OR “sickle cell anaemia” OR “sickle anemia” OR “sickle anaemia” OR drepanocytosis OR “hemoglobin s disease” OR “sickle cell disorder” OR “sickling disorder” OR “hbs disease” OR “sickle cell disease”) AND (DE stroke OR stroke OR strokes OR “Cerebrovascular Accident” OR “Cerebrovascular Accidents” OR Cerebrovascular Apoplexy OR CVA OR CVAs OR “Brain Vascular Accident” OR “Brain Vascular Accidents” OR “brain attacks” OR “brain accident” OR “brain blood flow disturbance” OR “brain blood flow disturbances” OR “brain accidents” OR “brain insult” OR “brain insults” OR “cerebral attack” OR “cerebral attacks” OR “cerebral accident” OR “cerebral accidents” OR “cerebral blood flow disturbance” OR “cerebral blood flow disturbances” OR “cerebral insult” OR “cerebral insults” OR “cerebral vascular insufficiency” OR “cerebrovascular attack” OR “cerebrovascular attacks” OR “cerebrovascular accident” OR “cerebrovascular accidents” OR “cerebrovascular blood flow disturbance” OR “cerebrovascular blood flow disturbances” OR “cerebrovascular insult” OR “cerebrovascular insults”) AND ((DE “genetic markers” OR “genetic marker” OR “genetic markers” OR “genetic biomarker” OR “genetic biomarkers” OR “dna marker” OR “dna markers” OR “chromosome marker” OR “chromosome markers”) OR (BCL11A) OR (“transforming growth factor beta receptor 3”/exp OR TGFBR3 OR “beta-glycan” OR betaglycan OR “TGF-beta type III” OR “TGF-beta type 3” OR “TGF-beta receptor type III” OR “TGF-beta receptor type 3” OR “transforming growth factor beta type III” OR “transforming growth factor beta type 3” OR VCAM1) OR (“adenylate cyclase 9” OR “adenylyl cyclase IX” OR ADCY9 OR “adenylyl cyclase type IX”) OR (ANXA2 OR “annexin A2 protein” OR “annexin 2”) OR (TEK OR “TIE-2” OR tie2 OR “angiopoietin-1 receptor” OR “angiopoietin receptor”) OR (“adenylate cyclase 9” OR “adenylyl cyclase IX” OR “adenylyl cyclase type IX” OR ADCY9) OR (“Vascular Cell Adhesion Molecule 1” OR “Inducible Cell Adhesion Molecule 110” OR “CD106 Antigens” OR “VCAM 1” OR “CD106 antigen” OR “Vascular Cell Adhesion Molecule” OR “INCAM 110”) OR (“ectonucleotide pyrophosphatase phosphodiesterase 1” OR ENPP1 OR “ecto-nucleotide pyrophosphatase phosphodiesterase 1” OR “glycoprotein PC-1” OR “plasma cell membrane glycoprotein PC-1” OR “alkaline phosphodiesterase 1” OR NPP1 OR “major aFGF-stimulated phosphoprotein” OR “PC-1 glycoprotein” OR “plasma-cell membrane glycoprotein 1” OR “MAPF protein” OR “major acidic fibroblast growth factor-stimulated phosphoprotein”) OR (“plasma-cell membrane glycoprotein 1” OR “Glucosephosphate Dehydrogenase” OR “6 phosphate dehydrogenase” OR “6 phosphoglucose dehydrogenase” OR “Glucose 6 Phosphate Dehydrogenase” OR “6 phosphoglucoside dehydrogenase”) OR (DE “alpha Thalassemia” OR “alpha-Thalassemia” OR “alpha thalassaemia” OR “Thalassemia alpha” OR “a thalassemia” OR “Hemoglobin H Disease”) OR (hbg2) OR (BCL11A OR “C2H2-type zinc finger protein” OR “B-cell CLL-lymphoma 11A” OR “CTIP1 protein”))
